# Cellular resilience and baboon aging

**DOI:** 10.18632/aging.203728

**Published:** 2021-11-29

**Authors:** Daniel A. Adekunbi, Peter W. Nathanielsz, Adam B. Salmon

**Affiliations:** 1 Texas Pregnancy and Life-Course Health Research Center, Department of Animal Science, University of Wyoming, Laramie, WY 82071, USA; 2 Barshop Institute for Longevity and Aging Studies, The University of Texas Health Science Center at San Antonio, San Antonio, TX 78229, USA

**Keywords:** aging, fibroblast, baboon, oxidative stress, cell proliferation, resilience

“True stability results when presumed order and presumed disorder are in balance. A truly stable system expects the unexpected, is prepared to be disrupted, waits to be transformed” (Tom Robbins, American Novelist) [1].

The above quote resonates with an important concept in Biology – Homeostasis – the delicate balance of maintaining cellular and systemic function. The physiological processes that maintain homeostasis progressively decline with aging and lead to disease vulnerability. A major component of homeostasis is the coordinated dynamic response to restore stability following disturbances to normal conditions which can be otherwise described as resilience. At the cellular level, resilience is determined by cell responses to stressor(s) and their ability to recover from stress by mobilization of underlying biological reserve to achieve homeostasis. Stress conditions caused by several sources including free radicals, protein aggregation, nutrient deficit, or dysregulated neuroendocrine stress axis disrupt homeostasis and are thought to substantially contribute to aging [2,3]. Clarifying the cellular machinery responsible for cellular homeostasis in aging can lead to the identification of aging mechanisms and suggest possible therapeutic options to delay age-related diseases.

Several model systems have enhanced our understanding of aging processes, but they are limited by their translatability and direct applications to humans. Non-human primate (NHP) models serve a unique purpose of covering the translational gap in addressing fundamental questions on aging mechanisms. When coupled with cell culture approaches, NHP models are a powerful resource for longitudinal studies especially since they provide opportunity for multiple assessments of various biological endpoints using the powerful tools available for high throughput, highly specific mechanistic studies. Our recent paper used a cell culture system that involves primary skin-derived fibroblasts cultures obtained from male and female baboons across the life course (4 to 21 years; human equivalent 14 to 73 years) to characterize cellular responses to homeostatic challenges [4]. The challenges incorporated included those that directly impact some of the hallmarks of aging including oxidative stress (hydrogen peroxide (H_2_O_2_) and paraquat), proteostasis (thapsigargin), intracellular signaling (dexamethasone) and metabolic regulation (low glucose concentrations). Together these stressors provide a panel of resilience outcomes and establish a framework for assessing cellular resilience mechanisms.

**Figure 1:**
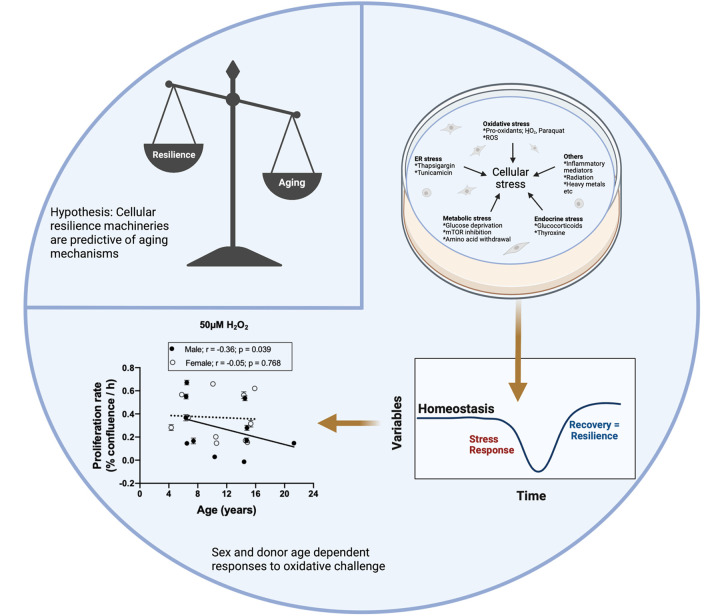
**Cellular proliferation responses to homeostatic challenges in primary skin-derived baboon fibroblasts.** Overview of baboon cell culture model developed to determine cellular resilience to homeostatic challenges. Representative data show age and sex modify cellular proliferation responses to oxidative challenge in Baboon fibroblasts. Figure created in biorender.com

Because resilience tests the ability to respond to challenge, tests that measure cellular resilience should be carefully designed to assess both a deviation from homeostasis and the kinetic response towards return. We have used changes in proliferation rate as a functional readout of cell response to an acute challenge. Leveraging a live-cell imaging system, the kinetics of cell proliferation in terms of response to challenge and return to homeostasis can be addressed. Our study demonstrated that cells treated with low dose H_2_O_2_ (50 µM) show an impairment of proliferation during and immediately after the challenge, followed by resumption in proliferation albeit at a reduced rate [4]. Remarkably, the ability of fibroblasts to return to proliferation following H_2_O_2_ or paraquat challenge was significantly associated with donor age in cells from male baboons but not in those from females (Figure 1). This sexual dimorphism lends credence to diminished cellular resilience with age and further establishes sex-related differences in resilience outcomes, particularly in response to oxidative stress [5]. Similarly, metabolic stress induced by low glucose significantly impaired proliferation of fibroblasts from old donors of both sexes compared to young donors, indicating increased metabolic demands of the older cell lines and their dependence on glycolysis for survival. However, our study also highlights that the increasing donor age is not always a detriment to response to cellular challenge. Responses to thapsigargin-induced ER stress were also age and sex dependent, though with different pattern than that of oxidative challenge. In this case, young females alone were vulnerable to ER stress, while young males and old females were not affected. Resilience to glucocorticoid challenge was also age and sex dependent in a similar pattern; young female-derived fibroblasts were less resilient to dexamethasone while young males and old donors of both sexes had no impairment. Whether these results are related to the permeability of baboon fibroblasts to dexamethasone or divergent pathways are activated in response to specific stressors (e.g oxidative versus glucocorticoid stress) warrants further investigation. Further, the sex effects we note are likely to be related to the specific sexual environment of the donor, since the culturing procedure for both male and female-derived cells were identical, including similar estrogen concentrations in the culture media. The study further highlights the propagation of sexual dimorphism in cell lines which unfortunately has been largely ignored in most studies [6].

In conclusion, we highlight the development of a novel model to measure cellular resilience and its relationship to NHP aging. With this model, we have clearly identified age- and sex- dependent response to cellular challenge in aging baboons. Moreover, this model can be used to clarify the underlying mechanisms mediating resilience as well as explore whether cellular resilience can be indicative of healthy aging and longevity. Clarifying these mechanisms then will significantly strengthen the ability to improve health span and healthy aging in translatable ways.
